# Community case management to accelerate access to healthcare in Mali: a realist process evaluation nested within a cluster randomized trial

**DOI:** 10.1093/heapol/czae066

**Published:** 2024-07-26

**Authors:** Caroline Whidden, Amadou Beydi Cissé, Faith Cole, Saibou Doumbia, Abdoulaye Guindo, Youssouf Karambé, Emily Treleaven, Jenny Liu, Oumar Tolo, Lamine Guindo, Bréhima Togola, Calvin Chiu, Aly Tembely, Youssouf Keita, Brian Greenwood, Daniel Chandramohan, Ari Johnson, Kassoum Kayentao, Jayne Webster

**Affiliations:** Department of Disease Control, London School of Hygiene and Tropical Medicine, Keppel Street, London WC1E 7HT, United Kingdom; Department of Research, Monitoring & Evaluation, Muso, SEMA Route de 501 Logements, Bamako H3M8+VJC, Mali; Muso, SEMA Route de 501 Logements, Bamako H3M8+VJC, Mali; Department of Anthropology, University of California, 375 Portola Plaza, Los Angeles, CA 90095, United States; Institute for Social Research, University of Michigan, 426 Thompson Street, Ann Arbor, MI 48103, United States; Department of Research, Monitoring & Evaluation, Muso, SEMA Route de 501 Logements, Bamako H3M8+VJC, Mali; Faculté des Sciences de l’Éducation et des Sciences Humaines, Université des Lettres et des Sciences Humaines de Bamako, Rue 627 Porte 83, Bamako BP E 2528, Mali; Institut National de la Jeunesse et des Sports, Bamako J35J+CJF, Mali; Institute for Social Research, University of Michigan, 426 Thompson Street, Ann Arbor, MI 48103, United States; Institute for Health & Aging, University of California, 490 Illinois Street, San Francisco, CA 94158, United States; Muso, SEMA Route de 501 Logements, Bamako H3M8+VJC, Mali; Department of Research, Monitoring & Evaluation, Muso, SEMA Route de 501 Logements, Bamako H3M8+VJC, Mali; Department of Research, Monitoring & Evaluation, Muso, SEMA Route de 501 Logements, Bamako H3M8+VJC, Mali; School of Public Health, University of California, 2121 Berkeley Way, Berkeley, CA 94704, United States; Ministère de la Santé et du Développement Social, Cité Administrative, Bamako JXGR+R48, Mali; Muso, SEMA Route de 501 Logements, Bamako H3M8+VJC, Mali; Department of Disease Control, London School of Hygiene and Tropical Medicine, Keppel Street, London WC1E 7HT, United Kingdom; Department of Disease Control, London School of Hygiene and Tropical Medicine, Keppel Street, London WC1E 7HT, United Kingdom; Muso, SEMA Route de 501 Logements, Bamako H3M8+VJC, Mali; Institute for Global Health Sciences, University of California, 550 16th Street, San Francisco, CA 94110, United States; Department of Research, Monitoring & Evaluation, Muso, SEMA Route de 501 Logements, Bamako H3M8+VJC, Mali; Malaria Research & Training Centre, Université des Sciences, des Techniques et des Technologies de Bamako, Bamako PO Box 1805, Mali; Department of Disease Control, London School of Hygiene and Tropical Medicine, Keppel Street, London WC1E 7HT, United Kingdom

**Keywords:** Process evaluation, health services research, health systems evaluation, primary health care, community health workers, realist evaluation, cluster randomized trials

## Abstract

The Proactive Community Case Management (ProCCM) trial in Mali reinforced the health system across both arms with user fee removal, professional community health workers (CHWs) and upgraded primary health centres (PHCs)—and randomized village-clusters to receive proactive home visits by CHWs (intervention) or fixed site-based services by passive CHWs (control). Across both arms, sick children’s 24-hour treatment and pregnant women’s four or more antenatal visits doubled, and under-5 mortality halved, over 3 years compared with baseline. In the intervention arm, proactive CHW home visits had modest effects on children’s curative and women’s antenatal care utilization, but no effect on under-5 mortality, compared with the control arm. We aimed to explain these results by examining implementation, mechanisms and context in both arms We conducted a process evaluation with a mixed method convergent design that included 79 in-depth interviews with providers and participants over two time-points, surveys with 195 providers and secondary analyses of clinical data. We embedded realist approaches in novel ways to test, refine and consolidate theories about how ProCCM worked, generating three context-intervention-actor-mechanism-outcome nodes that unfolded in a cascade. First, removing user fees and deploying professional CHWs in every cluster enabled participants to seek health sector care promptly and created a context of facilitated access. Second, health systems support to all CHWs and PHCs enabled equitable, respectful, quality healthcare, which motivated increased, rapid utilization. Third, proactive CHW home visits facilitated CHWs and participants to deliver and seek care, and build relationships, trust and expectations, but these mechanisms were also activated in both arms. Addressing multiple structural barriers to care, user fee removal, professional CHWs and upgraded clinics interacted with providers’ and patients’ agency to achieve rapid care and child survival in both arms. Proactive home visits expedited or compounded mechanisms that were activated and changed the context across arms.

Key messages

- The WHO recommends health policy and system support to optimize community health worker (CHW) programmes, but evidence gaps persist to recommend specific interventions, including how to deliver CHW services, the role of context and implications of implementing multi-component CHW interventions.
- This study brings together different research methodologies in new ways to enhance a process evaluation and accommodate the complexity inherent in community health systems interventions.
- Within a cluster randomized trial, we demonstrate how proactive CHW home visits accelerated maternal and child healthcare utilization via mechanisms that were also activated by health system support co-interventions in both arms of the trial, which had changed the context within which the home visit intervention was implemented.
- By addressing multiple structural barriers to care, user fee removal, professional CHWs and upgraded primary care clinics in both trial arms interacted in complex ways with providers’ and patients’ agency to achieve rapid care and child survival across arms over 3 years, despite the onset of armed conflict.

## Introduction

Governments around the world are scaling up community health worker (CHW) programmes to improve service coverage and health outcomes (Hodgins *et al*., 2021). Further research is needed to understand how CHWs can be integrated into, and supported by, health systems and communities (World Health Organization, 2018). Specifically, research is needed on how to organize CHW workflows and approaches to deliver CHW services that optimize impact (World Health Organization, 2018).

Proactive Community Case Management (ProCCM) is a multi-component intervention based on formative research that identified financial, health system and social barriers to care in periurban Mali (Johnson *et al*., 2012). ProCCM includes (Johnson *et al*., 2018):

- Proactive home visits: CHWs conduct routine door-to-door home visits, identifying prospective patients and proactively offering promotive, preventive and curative care at patients’ doorsteps.
- Professional CHW care: CHWs are salaried, trained and supervised to provide comprehensive primary healthcare in communities, including reproductive, maternal and integrated Community Case Management services (Young *et al*., 2012).
- Reinforced primary care clinics: public sector primary health centres (PHCs), to which CHWs refer cases outside their scope, receive improvements in infrastructure, equipment, supplies, recruitment and training.
- User fee removal: all fees are removed at all points of care, including ambulatory evacuation and care at secondary or tertiary referral hospitals.

We conducted the ProCCM trial (Supplementary Figure S1) in Bankass, Mali, from February 2017 to April 2020. This cluster randomized trial had two arms, and both received ProCCM components two to four listed above. In the intervention arm only, village-clusters received proactive CHW home visits (2 hours per day, 6 days per week). In the control arm, village-clusters received ProCCM without component one listed above, where CHWs provided care exclusively at a community health site (4 hours per day, 6 days per week). We designed the trial to isolate a single component of ProCCM, proactive home visits by CHWs and assess its effectiveness to reduce under-5 mortality (primary endpoint) and increase child, maternal and reproductive healthcare utilization (secondary endpoints) compared with a fixed site-based approach to CHW service delivery (Whidden *et al*., 2019). We also assessed trial outcomes across both arms over time, comparing the 3-year implementation period to the baseline period.

Between trial arms, we found no difference in the incidence rate of under-5 mortality (Liu *et al*., 2024). After 12 months, sick children had 22% higher odds of prompt (24-hour) treatment from the health sector in intervention compared with control clusters [95% Confidence Intervals (CIs): 1.06, 1.41], but no difference at 24 or 36 months (Whidden *et al*., 2023). Over all 3 years, we found some evidence that home visits increased children’s health sector consultation (Odds Ratio = 1.12; 95% CIs: 0.99, 1.26). We found no difference between arms in institutional delivery, although pregnant women were 11% more likely to initiate antenatal care (ANC) in the first trimester (95% CIs: 1.02, 1.19), and 25% more likely to receive four or more ANC visits (95% CIs: 1.08, 1.43) in intervention compared with control clusters (Kayentao *et al*., 2023).

Across trial arms, we found marked improvements in child survival and healthcare utilization compared with the baseline period, despite the escalation of armed conflict. Under-5 mortality reduced by more than 60%, from 148.4 to 55.1 deaths per 1000 live births (Liu *et al*., 2024), and sick children’s prompt treatment more than doubled (Whidden *et al*., 2023). Any ANC increased by 83% (95% CIs: 1.78, 1.86), first trimester ANC by 15% (95% CIs: 1.06, 1.25), four or more ANC visits by 2.59 times (95% CIs: 2.28, 2.91) and institutional delivery by 54% (95% CIs: 1.41, 1.66), compared with baseline (Kayentao *et al*., 2023).

We embedded a process evaluation to explain the results of the trial of the home visit intervention and to determine whether and how ProCCM as a whole could be effective in a rural and remote Malian context. Guided by the process evaluation framework of the UK’s Medical Research Council (Moore *et al*., 2015) and an adaptation for cluster trials (Grant *et al*., 2013), the ProCCM process evaluation thus examined implementation, mechanisms and context in both arms of the ProCCM trial. This is the process evaluation of a health system intervention (ProCCM) in the context of a trial that quantified the impact of the service delivery component (home visits) of that system.

## Methods

### Study design

We conducted a mixed method process evaluation with a convergent design, in which we collected and analysed quantitative and qualitative data separately, then compared and interpreted the results together (Creswell and Plano Clark, 2018). Data sources included a close-ended survey with providers (CHWs, CHW supervisors and PHC staff), two rounds of qualitative in-depth interviews (IDIs) with trial providers and participants (community members) and clinical data collected by CHWs and PHCs.

We embedded realist approaches within this process evaluation conducted alongside a cluster randomized trial (Bonell *et al*., 2012), because these methods have been developed precisely to scrutinize how, why, for whom and in what contexts complex interventions work (Pawson and Tilley, 1997). At different stages in the evaluation, we used both Theory of Change (ToC) and Realistic Evaluation approaches (Blamey and Mackenzie, 2007). We started with a ToC logic model depicting an implementation theory that linked ProCCM’s activities to intended outcomes, which we workshopped with programme designers and managers. We then used the ToC to map what mixed method data to assemble, and complimented it with realist approaches in data collection, analysis, integration and interpretation. This allowed us to iteratively test, refine and consolidate programme theories that linked ProCCM’s causal mechanisms and context to outcomes, which we report as context-intervention-actor-mechanism-outcome (CIAMO) configurations (Hamon *et al*., 2020).

### Study site

The study was conducted in seven contiguous, rural health catchment areas home to approximately 100 000 people, each serviced by a public sector PHC, in the Bankass district in central Mali. PHCs are managed by Community Health Associations (ASACO), elected committees of local community members, and linked to the district referral hospital outside the study area. At baseline, 17 CHWs (*agents de santé communautaires*) stationed at fixed sites serviced some villages greater than 5 kilometres from a PHC and worked with community health volunteers (*relais communautaires*) who engaged in health education, promotion and mass distribution campaigns. Prior to ProCCM, CHWs and PHCs charged user fees to care-seeking patients. Healthcare utilization and under-5 mortality were worse in this setting at baseline than national and regional averages (Boettiger *et al*., 2021; Treleaven *et al*., 2021; Whidden *et al*., 2021).

Approximately 1 year into the ProCCM trial, armed conflict spread and intensified in central Mali (Human Rights Watch, 2020), affecting the lives of trial providers and participants. Minority communities enrolled in the trial (four entire clusters and 10 partial clusters) were destroyed or displaced. Starting in December 2018, we adapted the programme in nine of the 137 clusters to mitigate the security risks in accessing or delivering services, by deploying a mobile PHC clinic and/or relocating CHWs who travelled into their clusters.

### Data collection

#### Providers’ survey

We developed a short, structured questionnaire that covered health worker characteristics. We administered the survey during the trial period (April, May 2019) to all CHWs (*N* = 168) and dedicated CHW supervisors (*N* = 10); we added PHC workers (*N* = 20), including technical directors, maternity ward providers and pharmacists, after the trial period (November 2020). We administered the survey at a place of work in French or Bambara, depending on the respondent’s choice.

#### IDIs

We conducted a total of 79 IDIs over two time-points, at a mid-line point during the trial (July 2019) and at an endline point after the trial (August 2020), with different respondents to explore changes over time and glean and refine theories about how ProCCM worked (Manzano, 2016). At each of the two qualitative data collection rounds, we selected a purposive sample of CHWs (*N* = 12), CHW supervisors (*N* = 5), PHC providers (*N* = 4) and trial participants (*N* = 15). Within each respondent type, we sampled to ensure variability in gender, geography and trial arm (CHWs) or role (in the PHC, community or household). Respondent availability, insecurity and road conditions limited access to some targets; thus, we added seven interviews in January 2021 with CHWs (*N* = 2) and female participants (*N* = 5), all from geographically remote clusters.

Prior to each qualitative data collection round, we developed a semi-structured qualitative interview guide for each respondent type that we piloted outside the study area. Mid-line interview guides asked respondents to share experiences and perspectives about the programme and its outcomes, mechanisms and context. Endline interview guides incorporated realist interviewing techniques, where tentative theories about how ProCCM worked were presented to respondents, eliciting reactions and stories to refine programme theories (Manzano, 2016). Two Malian, male anthropologists who were not from the study area or part of the trial or implementation teams conducted IDIs in French or Bambara or, if this was not possible, an interpreter (also not from the trial area or team) provided translation in real time via a local language. Interviews lasted between 45 and 120 minutes; longer interviews tended to be those requiring translation or with supervisor respondents. All interviews were audio-recorded and transcribed in French.

#### Clinical data

PHCs collected patient data in paper registers, which were aggregated monthly and entered into the District Health Information Software II (DHIS2). We extracted PHC-month-level count data on facility service utilization approximately 1 year before and 3 years during the trial. CHWs collected patient data during routine encounters, including proactive home visits, on a mobile phone application (Community Health Toolkit). We extracted de-identified encounter-level data on CHW service utilization in both trial arms.

### Analysis

We coded qualitative data using a hybrid deductive and inductive approach to thematic analysis. We developed an initial hierarchical coding frame based on the evaluation’s aims and frameworks, which we revised and supplemented based on themes that emerged in the data. Three investigators independently coded the same five mid-line and endline interviews. Two investigators divided the remaining transcripts equally, coding all interviews using NVivo 12 (QSR International, 2017). Coders maintained personal reflexive journals and met weekly to ensure intra and intercoder consistency, iteratively update the coding frame and share reactions to data excerpts or patterns in the dataset. In addition to interview summaries, coders wrote analytic memos to capture emerging ideas or higher level thinking while coding (Miles *et al*., 2013).

Once the mid-line dataset was coded, we consolidated analytic memos into propositions or initial programme theories. We iteratively tested and refined our theories using realist retroduction that moves back and forth between inductive and deductive logic (The RAMESES II Project, 2017; Gilmore *et al*., 2019), including discussions with programme managers and researchers, realist interviews with providers and trial participants and interrogating quantitative data. We descriptively analysed provider survey, CHW application and DHIS2 data using Stata 15 (StataCorp, 2017), Stata 17 (StataCorp, 2021) and Excel (Microsoft Corporation, 2021), respectively.

We compared mixed methods evidence against these emerging theories to see whether it reaffirmed, reshaped or contradicted our understanding. We generated three CIAMO nodes that each include multiple contextual factors (C), intervention components (I), actors (A), mechanisms (M) and/or outcomes (O) that act inter-dependently reflecting the complex analytic reasoning that people engage in when they interact with health system interventions. These nodes relate to each other in a cascade (Webster *et al*., 2021), as each one triggered mechanisms and/or led to outcomes that changed the context within which the next node operated. The first two nodes encompass CIAMOs that were present in both arms of the trial to explicate how and why changes occurred in both arms relative to baseline. The third node contains CIAMOs specific to proactive CHW home visits to explicate the effects and null effects in the intervention arm relative to the control, in the context engendered by the first two CIAMO nodes.

## Results

Providers had a median age of 26 years (Table 1). More than half (58%) of CHWs were female, and almost all were from the village (44%), district (29%) or region (7%) within which they were deployed. Three supervisors (30%) and 20 CHWs (12%) had previous work experience or training in health. CHW characteristics were similar between arms.

**Table 1.** Socio-demographic and work-related characteristics of trial providers

			CHW		
	PHC provider, *N* = 20	Supervisor, *N* = 10	Intervention, *N* = 82	Control, *N* = 83	Total, *N* = 195
	*n* (%)	*n* (%)	*n* (%)	*n* (%)	*n* (%)
Age					
Median (IQR)	30 (26.5, 36.5)	32 (28, 36)	25 (23, 28)	26 (24, 28)	26 (24, 29)
Sex					
Male	10 (50)	8 (80)	36 (44)	34 (41)	88 (45)
Female	10 (50)	2 (20)	46 (56)	49 (59)	107 (55)
Education					
Primary (years 1–9)	2 (10)	0 (0)	13 (16)	16 (19)	31 (16)
Secondary (years 10–12)	10 (50)	3 (30)	66 (80)	63 (76)	142 (73)
Higher education	8 (40)	7 (70)	3 (4)	4 (5)	22 (11)
Marital status					
Not married	4 (20)	2 (20)	20 (24)	13 (16)	39 (20)
Polygynous	5 (25)	4 (40)	17 (21)	22 (26)	48 (25)
Monogamous	11 (55)	4 (40)	45 (55)	48 (58)	108 (55)
Household size					
Median (IQR)	4.5 (3, 6.5)	1 (1, 4)	4 (3, 6)	4 (3, 6)	4 (3, 6)
Religion					
Muslim	19 (95)	9 (90)	67 (82)	74 (89)	169 (87)
Christian	1 (5)	1 (10)	15 (18)	9 (11)	26 (13)
Cultural origin					
Dogon	10 (50)	5 (50)	79 (96)	76 (92)	170 (87)
Other	10 (50)	5 (50)	3 (4)	7 (8)	23 (12)
Relocated to catchment area					
Born/before trial	6 (30)	1 (10)	40 (49)	32 (39)	79 (41)
For trial from within district^a^	4 (20)	2 (20)	24 (29)	24 (29)	54 (28)
For trial from within region^a^	2 (10)	2 (20)	4 (5)	8 (10)	16 (8)
For trial from outside region^a^	8 (40)	5 (50)	2 (2)	0 (0)	15 (8)
Missing	0 (0)	0 (0)	12 (15)	19 (23)	31 (16)
Engages in other paid work^b^	1 (5)	2 (20)	11 (13)	13 (16)	27 (14)
Previous work experience^c^ or training in health prior to trial	18 (90)	3 (30)	10 (12)	10 (12)	36 (19)
Current/ongoing stockout^d^	2 (10)	8 (80)	73 (89)	70 (84)	145 (78)
Mean (min, max) weeklong stockouts^d^^,^^e^ since trial launch	1.3 (0, 3)	1.9 (0, 4)	1.2 (0, 3)	1.1 (0, 3)	1.2 (0, 4)
Mean (SD) clinical protocol knowledge score (max 19)	17 (2.1)	NA	16 (1.5)	16 (1.9)	16 (1.8)
Mean (SD) gender norms and attitudes scale^f^ (max 14)	12.4 (1.1)	12.2 (1.4)	12.2 (1.2)	12.2 (1.4)	12.2 (1.3)
Mean (SD) work days per week	6.4 (0.5)	3.0 (1.1)	5.6 (2.1)	5.8 (0.8)	5.6 (1.2)
Mean (SD) work hours per day	8.2 (0.9)	6.3 (1.4)	4.0 (0.7)	4.0 (0.6)	4.6 (1.6)
Mean (SD) times contacted by patients the previous work day	2.0 (2.6)	NA	2.6 (3.8)	2.9 (4.3)	2.7 (3.9)

Note(s): Characteristics are at the time of the survey (May–April 2019 for CHWs and supervisors, November 2020 for PHC).

a From within the Bankass health district, or within or outside the Mopti region.

b Two-thirds of CHWs who reported other paid work were women and they reported small business activities (*commerce*) or housework; men were involved in *commerce* or herding.

c This includes the 17 CHWs at baseline who are all ProCCM CHWs.

d CHWs reported vitamin A and artesunate suppository stockouts; supervisors reported a vitamin A stockout.

e Five out of 14 PHC providers who reported a stockout specified an antimalarial.

f Higher scores are more egalitarian (source: Waszak *et al*., 2001).

### CIAMO node 1: PHC and CHW care available without fees enabled care-seeking without delay

In the prevailing health system context [user fees, distance to PHC, insecurity, poverty and gender inequality or gendered social norms (C1)], removing user fees and deploying salaried CHWs linked to the formal health system (I) immediately led to more universal, frequent and rapid public sector care-seeking (O) by expanding the healthcare options readily available to participants and empowering them, especially women (A), in their ability to make strategic choices and act on their healthcare needs and desires (M) (Table 2). This CIAMO node was activated in both trial arms, fundamentally changing the context in which healthcare was delivered and received (C2).

**Table 2.** Context-intervention-actor-mechanism-outcome nodes

CIAMO node 1	In the prevailing health system context [user fees, distance to PHC, insecurity, poverty and gender inequality or gendered social norms (C1)], removing user fees and deploying salaried CHWs linked to the formal health system (I) immediately led to more universal, frequent and rapid public sector care-seeking (O) by expanding the healthcare options readily available to participants and empowering them, especially women (A), in their ability to make strategic choices and act on their healthcare needs and desires (M).
Context	Public-sector user fees Poverty, rural setting Intensive labour/time-constrained agricultural livelihoods High absolute poverty and wealth tied up in assets, e.g. animals Donkey cart transportation, wealthier households may have motorcycle Remote Median distance to nearest PHC of 6 kilometres (min <1, max >12) Poor road conditions, some cliffs and rivers Insecurity Unsafe to travel, especially after dark Temporary laws against motorcycle transport Gender inequality and/or gendered social norms Women ask male heads of household for money, transport and permission to seek care for their own and their children’s health Women’s labour burden/time poverty, including household chores, caregiving, agriculture, commerce
Interventions	User fee removal Salaried CHWs in every cluster, integrated within the formal health system Referral system, including ambulatory service
Mechanisms and actors	Participants’ ability to choose public-sector care among the care options affordable and available to them Participants’ ability to act quickly on their wants/needs, without having to assemble the means to pay or reach public-sector care Women’s ability to seek care more autonomously Participants’ social networks: other family members’ ability to support, encourage or participate in women’s and children’s care-seeking
Outcomes	Facilitated access to public-sector care Removed direct costs and reduced distance and indirect (transport, time, opportunity) costs Improved affordability, availability (proximity) and accommodation of public-sector care Increased public-sector utilization and prompt utilization More universal, frequent and faster curative care-seeking from within the health sector More and earlier maternal care-seeking from within the health sector, including ANC and institutional delivery Health and wellbeing Less suffering Fewer child deaths Empowerment (ability to make strategic life choices related to health) Less conflict, more social cohesion Less poverty, more resources (money, time) to invest elsewhere
CIAMO node 2	In the context of facilitated access and increased, rapid utilization (established by CIAMO node 1) (C2), upgraded PHC and professional CHW support in both trial arms (I) motivated more universal and rapid healthcare utilization and engendered new care-seeking norms (O) as providers and patients (A) built relationships, trust, expectations and social networks (M) through a mutually acceptable, quality experience delivering and receiving care (C3).
Context	Poverty, rural, remote, insecurity, gendered inequality and/or social norms (C1) Facilitated access to public-sector care created by CIMAO node 1 (C2)
Interventions	Upgraded PHCs Financing (user fee removal, reliable HW salaries) Infrastructure, equipment and supply chain Recruitment (including a mid-wife) and training Referral system to hospital care Professional CHWs Financing (user fee removal, reliable HW salaries) Stocks and supply chain Recruitment, training and dedicated supervision Referral system to PHC care
Mechanisms and actors	Patients felt they were treated equitably at reception and with dignity Providers’ ability to provide care and self-efficacy (feeling they were able to do what they needed to do) Providers’ motivation due to system resources and patients’ utilization/gratitude Mutual respect and relationship building between providers and patients Participants’ trust and expectations in the health system Participants’ social networks circulated motivating examples
Outcomes	Improved acceptability and quality of healthcare More universal, frequent and faster curative care-seeking (and treatment adherence) from within the health sector More and earlier maternal care-seeking from within the health sector, including ANC and institutional delivery Improved health knowledge, disease prevention and symptom recognition
CIAMO node 3	In an accessible, quality health system context (established by CIAMO nodes 1 and 2) (C3), proactive CHW home visits (I) prompted slightly more and earlier utilization in the intervention arm (O) by enabling participants’ and providers’ (A) abilities to seek and deliver services, and to build relationships, mutual trust, expectations and social networks (M), but these mechanisms were already activated in both trial arms
Context	Poverty, rural, remote, insecurity, gender inequality and/or gendered social norms and social values towards the elderly (C1) Facilitated access to public-sector care created by CIMAO node 1 (C2) Acceptable, quality public-sector care created by CIAMO node 2 (C3) Increased, rapid health service utilization created by CIAMO nodes 1 and 2
Interventions	Proactive CHW home visits
Mechanisms and actors	Perceived opportunity cost and ability to reach public-sector care Participants felt accommodated and respected when treated at home CHWs’ ability to deliver promotive, preventive and follow-up services CHWs’ and participants’ perceptions of the CHW’s role/responsibility Relationship building and community embeddedness Participants’ trust and expectations in the health system CHWs ability to more actively participate in social networks
Outcomes	Improved accommodation and acceptability of healthcare Trust relationships and embeddedness between CHW and community Slightly more heath sector care utilization among sick children Slightly faster curative care-seeking among sick children, especially initially More and earlier ANC, including community ANC contacts More complete follow ups after treatment/referral Improved health knowledge, disease prevention and symptom recognition

**Figure 1.**
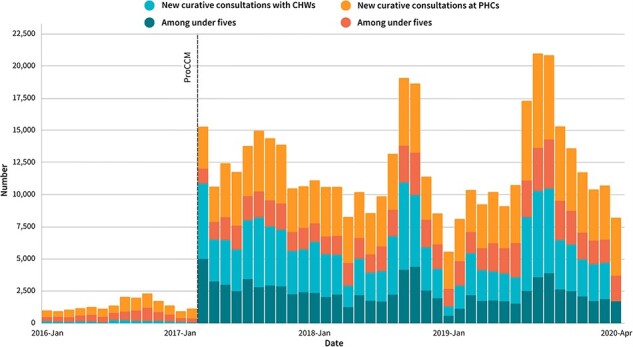
Number of PHC and CHW new curative consultations during the 14 months prior to ProCCM launch and the trial period

Notes: New curative consultation refers to sick patient assessments/diagnostic visits with CHWs or PHCs. PHC counts were derived from DHIS2 and CHW counts from the CHW application (except for the CHW counts prior to ProCCM which came from district health quarterly reports). Consultations with sick under 5 year olds are layered over top of the totals in a darker shade. A patient who was assessed by a CHW and referred to the PHC (and completed that referral) would be included in both PHC and CHW counts. No other follow-up visits were included at either CHW or PHC level.

Previously, due to user fees and distance, participants recalled having ‘no choice’ other than to wait to seek care from the public health system when faced with illness. They would first see if symptoms resolved on their own, ‘*se débrouiller*’ (manage) with traditional medicines and/or mobilize sufficient resources to reach and receive PHC care. A female control arm participant contextualized people’s care-seeking ‘preferences’ prior to the programme: ‘people had difficulty paying for care, which is why they preferred to heal the sick with traditional medicines, without any guarantee they would improve, than to travel kilometres for care they could not afford’ (#41-endline). In the first month of implementation, CHWs recorded over 10 000 sick patient diagnostic assessments, and PHCs registered over four times as many initial curative consultations with sick patients compared with the previous month (Figure 1). Overall, new curative consultations with public sector providers increased by 8.8 times, comparing the trial period to the 14 months prior (Figure 1). Participants reported that care had become ‘easier’ to access because there were no fees and CHW services were available close to or at home, enabling participants to choose care from within the public health system as a first recourse. A village chief in an intervention cluster explained: ‘nowadays, we have CHWs in the villages and *dogotorow* [providers] in the PHCs and all the care is free, so people no longer stay a long time at home with their illness’ (#19-endline).

Removing user fees and deploying CHWs in every cluster enabled some women to take and act on decisions pertaining to their and their children’s health more autonomously and quickly. Whereas many women previously asked male heads of household for the means to reach and pay for health sector care, respondents reported that women could now seek care on their own, simply ‘inform’, or request only ‘accompaniment’ and/or transport. Female participants from intervention and control clusters, respectively, explained: ‘now, even if your husband is not there, you have the possibility to go to the health centre because it’s free. Plus, we benefit from certain services at home from our CHW’ (#27-endline). ‘If the husband is nearby, it would be good to inform him, this is normal. If not, the ideal is to go without informing him because […] some diseases require a quick intervention’ (#38-endline). Another participant described how no fees and a (fixed) CHW reduced treatment delays:

Before, when you got sick, you would tell your husband. He would respond clearly that there is no money to treat you. You could stay cloistered in your room during two, three days, even a week. Eventually, you would go to your parents’ house to get care. It was the same for the children, it was the mother who suffered alongside her child. But all these are bad memories for us. Now, once you get sick, you take a day to observe your condition. If it doesn’t improve, the next day you go to [CHW] to get care or a referral form. (#19-mid-line)

Women’s care-seeking autonomy depended on household relationships and structures, gendered power dynamics, distance to PHC and insecurity. Critically, in areas and moments of heightened insecurity, temporary laws prohibited motorcycles, and the PHCs’ moto-ambulances would not service villages after dark. These restrictions inhibited access to PHC services in important ways, including rapid referral to obstetric care. The chief of a remote village explained: ‘With this insecurity, at night people are afraid to go [to the health centre]. It’s especially the women that are affected. At night the motos can’t leave and if we call the ambulance, it also doesn’t come. To go by donkey cart is also difficult. […] At that moment when the situation was *chaud* [hot, meaning intense], people didn’t leave, so we couldn’t have the health we wanted’ (#16-mid-line).

Removing ANC fees (less than USD$2) doubled women’s first ANC visits at PHCs in the first month of implementation (Supplementary Figure S2). Over the trial period, first ANC visit was 23% higher on average compared with 14 months before (*P* < 0.001), when providers recalled being unable to convince many women to attend. They would conduct village outreach campaigns and ‘women would run and hide because money had to be taken’ (mid-wife, #35-endline). A male ASACO member and former *relais* recalled ‘we used to sensitize pregnant women to come to the centre for prenatal follow up, but they told us their husbands didn’t have the money. […] Now if a woman gets pregnant, she gets up of her own accord to come and see us’ (#18-endline).

According to providers and participants, user fee removal also had direct economic and social impacts. Respondents reported less ‘conflict’ or ‘*mankan*’ (noise) and more ‘cohesion’ or ‘*entente*’ (understanding) between couples and within families because they were no longer confronted with difficult decisions about healthcare expenses and could allocate more resources to feeding the family or supporting children. As a male control arm participant explained:

The standard of living has increased in the community. We are farmers, after the harvest we used to put the grain at the women’s disposal and that was it. In case of illness, we had no money to care for our wives and our children. This naturally created small conflicts within the couple. But all these problems are over […] Now, heads of families have no more healthcare worries. The children are well and the women are also able to do their small business activities. (#17-mid-line)

### CIAMO node 2: systems support enabled respectful, quality PHC and CHW care that motivated utilization

In the context of facilitated access and increased, rapid utilization (C2), upgraded PHC and professional CHW support in both trial arms (I) motivated more universal and rapid healthcare utilization and engendered new care-seeking norms (O) as providers and patients (A) built relationships, trust, expectations and social networks (M) through a mutually acceptable, quality experience delivering and receiving care (C3) (Table 2).

When participants sought and reached public sector healthcare, they experienced an intake reception that they perceived as ‘welcoming’, ‘organized’ and equitable, which ‘prevents frustration between people, discrimination, and encourages us to seek care’ (female control arm participant, #30-endline). This included having a comfortable place to wait, being consulted in order of arrival or urgency and receiving treatment or referral quickly and at no cost. Patients used to be seen based on who could pay, and thus, the poor used to experience delays or were denied care once they reached the clinic. ‘Nothing is more frustrating than seeing someone, who came to find you at the health centre, access care before you. If this happens to me, I will no longer return to that place unless I have no other choice’ (#19-endline). Now, ‘it is the [referral] forms that talk. There is no need to say “I have money” or “I am poor”. It’s by order of arrival’ (head of household, #22-endline). This was so important to participants that providers and ASACO members recalled having to explain initially why emergency cases jumped ahead of the queue, a practice that then became widely accepted. ‘Today, the most urgent cases are seen first. This does not affect human dignity, it has nothing to do with disrespect. But before, when you had no means, there was no respect, no dignity on human life’ (female *relais*, #29-endline).

Providers and participants reported ‘respect’, compassion and patience in their interactions with each other, which was enabled, according to providers themselves and ASACO members, by health system inputs, namely: financing (e.g. reliable salaries, user fee removal), infrastructure (e.g. reception), human resources (e.g. recruitment), equipment (e.g. ambulance) and stocks and supplies (e.g. reliable drugs). Even with five times more curative visits to PHCs (Figure 1), the programme offered the resources providers needed to feel supported, capable and proud in their ability to provide care and be accountable to their patients. An auxiliary mid-wife (*matrone*) explained:

Before, our health centres were not well equipped. This caused a lot of problems for us. Often, faced with certain situations, you would ask yourself how to manage. […] When you meet the patient she will say that you are not welcoming. But she doesn’t know all the problems you are going through. You are there wondering how to do your job, but she doesn’t see all that. […] Now that the [healthcare] workers are everywhere and we have equipment, our comportment has also changed. We are more welcoming now that we have everything we need to do our job. (#30-mid-line)

In this enabling environment, providers emphasized the importance of ‘*l’accueil*’ (the welcoming reception). For a PHC deputy technical director, ‘a patient well received is a patient half cured. A good reception incites other patients to come to the health centre’ (#34-endline). For a female fixed CHW, ‘when women come, I smile with them, I welcome them well, until we become intimate friends. This is how I instill confidence between them and me’ (#9-mid-line). A female participant experienced this: ‘the *dogotorow* [providers] receive us well and they respect us. Everything happens with transparency, in communicating with the patient. Before, […] the doctor would treat you without telling you what you were suffering from. But now, […] the doctor takes all his time to explain to you all about your illness’ (#36-mid-line).

Trust in the health system care was instilled over time as participants experienced services to be effective, as well as respectful, available and affordable. ‘When you manage to cure a person of their illness, they will trust you’ (proactive CHW, #6-endline). ‘At the beginning, no one believed in free care. We mistrusted the medicines that the CHWs proposed. But, as time went on, we realized that the treatments were not only free but effective. This is how the people started to adhere […] to the care offered by CHWs’ (#19-endline). Through their personal and shared experiences, participants came to expect respectful, rapid, effective care once reached, which encouraged care-seeking. ‘Everyone knows that if you go hunting today and find game, you’ll go back tomorrow. It’s the same thing. When people are well received at the health centre and the treatments are effective, they will go every time they are sick’ explained a CHW supervisor (#14-endline). They will also encourage others to go, such as this female control arm participant:

I took my sick child to the health centre and they gave me medicine and ‘peanut paste’ [Plumpy’Nut]. Some days later, my child’s condition improved significantly. Sometime later, I noticed the same signs in the child of a neighbour. Immediately, I suggested to her to take her child to the health centre to benefit from the same treatment. She took her child, he got the same treatment, and his condition improved. (#38-endline)

From traditional to health sector treatments, from delayed to rapid care-seeking, from home births to ANC and institutional delivery, were among the most common ‘surprising changes’ reported by respondents. Providers and participants explained that women now attended ANC ‘in great numbers’ (*matrone*, #36-endline) or ‘preferred to deliver at the health centre’ (female participant, #32-endline) because ‘they found their importance in it’ (#36-endline), or ‘as time went on, they realized the benefits’ (#32-endline). Stories about women who attended ANC and saw their baby on the ultrasound, or who did not attend ANC and had a complicated delivery, or who delivered at a PHC and received post-natal and newborn care ‘served as examples’ for other pregnant women, orienting them towards the health system.

PHC providers were encouraged by the increased utilization, which in turn provided opportunities to develop their skills and serve their community. ‘Before, […] I came to the maternity and patients didn’t come, or very little. Plus, our bosses were tapping us on the head telling us the ANC rate was low, while I was crumbling under the weight of the work. But now, women come for consultation, all the numbers are up, and I find this very motivating’ (#30-mid-line). ‘I can say that I have 55 namesakes. These are girls that came into my hands or who I helped the parents to deliver […] My husband also has at least ten namesakes because of me!’ (#36-endline).

### CIAMO node 3: proactive CHW home visits facilitated service delivery and utilization in an already facilitated context

In an accessible, quality health system context (C3), proactive CHW home visits (I) prompted slightly more and earlier utilization in the intervention arm (O) by enabling participants’ and providers’ (A) abilities to seek and deliver services, and to build relationships, mutual trust, expectations and social networks (M), but these mechanisms were already activated in both trial arms (Table 2).

**Figure 2.**
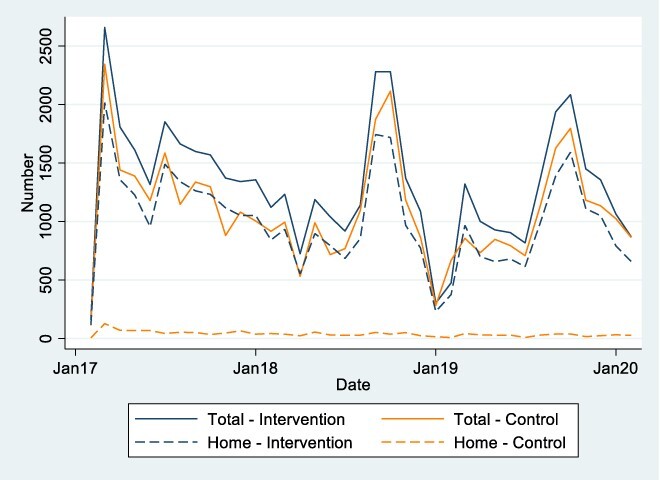
CHWs’ new curative consultations/diagnostic assessments with sick children under 5 by location (child’s home or accompanied by caregiver) by arm during the trial period

Source: CHW application. CHWs recorded the location of the sick under-5 patient assessment. Home refers to the child’s home. Other response options were accompanied by a parent, accompanied by a community member, and other.

From September 2017 to March 2020, intervention arm CHWs registered a median of 28 486 total home visits per month (205 per CHW per month), and control arm CHWs registered 2690 total per month (four per CHW per month) (Supplementary Figure S3; Supplementary Figure S4). Among new sick child consultations with CHWs, 76% occurred at the caregiver’s home in the intervention arm compared with 4% in the control arm, and the rest at the CHW’s site/home (Figure 2).

With a proactive CHW, participants appreciated that sick patients were ‘treated at home without having to travel’, which they found to be accommodating, respectful and confidential. Home visits ‘not only save us the trip, but also guarantees medical confidentiality’ (#31-endline) and ‘I find that the one that comes to you accords you an importance’ (#29-endline) reported two female intervention arm participants. Having heard about proactive CHWs in other villages, a male control arm participant liked ‘that you don’t tire yourself. Plus, when elders are sick, it is difficult to take them to the CHW. If the CHW could come to the house […] not everyone would see your sick patient’ (#17-mid-line). Participants in control clusters did not initially ‘accept’ the fixed workflow, and supervisors and PHC representatives were called in to defend it. Over time, control participants came to appreciate, and some prefer, the passive workflow because ‘at any moment we can find [CHW] at their site to treat certain illnesses that cannot wait’ (#37-endline) and expressed concern that ‘if the CHW was mobile, some people would surely find them absent’ (#41-endline). However, in both arms, participants reported that their CHW was available when needed, by phone or at home. The proactive CHW ‘does his rounds morning and evening. If someone is sick, they call him and he comes immediately’ (head of household, #35-mid-line).

Supervisors and CHWs in both arms believed that proactive CHWs ‘had more patients’ and ‘treated patients faster’. Proactive CHWs ‘discovered’ sick people during home visits who they believed would have otherwise waited to seek care or not sought care, while fixed CHWs were discouraged that people seemed to not seek care until the condition was more ‘serious’. For those with limited mobility, such as the very sick, the elderly and post-partum mothers and newborns, it could be ‘difficult’ to seek fixed site care. Those with labour burdens ‘might cancel their appointments with me to go to the field or tend to livestock’ (fixed CHW, #1-endline). ‘Because people have other occupations, they often wait until after work to come to the fixed CHW, and in the meantime the illness gets worse. Whereas proactive CHWs consult them even while they are working at home’ (fixed CHW, #12-endline). Many proactive CHWs adapted their home visit hours during the rainy season, so they would find women at home ‘pounding millet together’ (proactive CHW, #4-mid-line) rather than out in the fields. Among new sick child consultations recorded by CHWs, two-thirds (67%) in the intervention arm occurred the same/next day as symptom onset, compared with one-third (36%) in the control arm (Supplementary Figure S5). Furthermore, 28% in the intervention arm were diagnosed with danger or referral signs, compared with 38% in the control arm (Supplementary Figure S6). According to supervisors, fixed CHWs were ‘perceived as being there only to deliver the referral form’ or as gatekeepers to the PHC: ‘when sick people go to the fixed CHW, they often ask for the referral form [to the PHC] and not healthcare services for treatment or the medical visit. […] The fact that proactive CHWs conduct active case finding, it’s when the case exceeds their competence that they give the referral form’ (#14-endline).

A proactive CHW describes her responsibility as an active agent within the health system:

We, the proactive CHWs, cover the village searching and if we find a case, we don’t abandon them. Whereas fixed CHWs are immobile, as long as patients don’t come to them, they don’t go to patients. […] There are some pregnant women who don’t go to the health centre unless they fall sick. So, it’s up to us to go towards them, side by side, so that they come regularly to do their ANC. (#6-endline)

Home visits enabled proactive CHWs to better ensure patient follow up compared with fixed CHWs (Supplementary Figure S7), who ‘sensitized in vain that [patients] come for follow up. Tired, we left it alone’ (#10-endline). A male intervention arm participant reported ‘when [CHW] starts to treat a patient, he comes every day to see them until they are completely cured. […] When he starts to treat a child, he doesn’t leave him, deh! He follows him right up until the end of his treatment’ (#15-mid-line). However, some proactive CHWs reported challenges in finding their target patient during follow-up home visits.

Home visits helped CHWs build relationships, trust and embed within communities by inquiring about people’s health, ‘going toward’ the sick, following up, demonstrating the services on offer and counselling to promote health. Proactive CHWs were in ‘constant contact’ with their community and knew all the ‘worries’ and ‘secrets’ of the village. ‘It’s easier for a proactive CHW to gain someone’s trust since they communicate together every day, than a fixed CHW who people see only when they’re sick. Even if trust will establish between them, it will be slower than with proactive CHWs’ (female control arm participant, #25-endline). Through more regular and universal contacts (80% of CHW encounters with women were in the intervention arm), proactive CHWs could ‘encourage’ or ‘motivate’ care-seeking by reinforcing what participants could expect from the redesigned health system.

## Discussion

Central to the ProCCM trial, we hypothesized that CHW home visits would proactively detect sick patients and pregnant women, lead to earlier treatment and ANC initiation and thereby improve child survival and birth outcomes. Our process evaluation found that, while home visits may have accelerated access to care, ProCCM, regardless of CHW workflow, dismantled structural barriers to care that transformed the context in which we implemented and evaluated the home visit intervention. Together, user fee removal, professional CHWs and upgraded PHCs addressed direct costs, indirect costs (transport, time) and quality of healthcare, and interacted in multifaceted ways with people’s agency. Co-interventions in both trial arms enabled participants’ abilities and motivated their choices to seek care from within the public health system, resulting not only in more utilization but faster utilization, which is crucial for child survival and understudied in health policy and systems research.

Elimination of fees empowered participants when it came to healthcare, or activated ‘the process by which those who have been denied the ability to make strategic life choices acquire such an ability’ (Kabeer, 1999). With salaried, integrated CHWs in every cluster, public sector healthcare became as affordable and available as traditional or informal care, expanding the options with which participants could strategically engage. We saw large, immediate increases in maternal and children’s curative healthcare utilization, as seen in other user fee removal studies (Lagarde and Palmer, 2011). In our context, participants’ ‘capability space’—their choice, ability and opportunity (Frediani, 2010)—to seek affordable, available public sector care was influenced by the conflict, distance to PHC, gendered social norms and individual relationships. As experts on their body, their children and their context (Abimbola, 2023), participants navigated this space as ‘active patients’ (Leonard, 2014), seeking ProCCM services because they experienced them to be organized and fair, welcoming and respectful, rapid and effective. Financial, human and material resources enabled CHW and PHC providers’ ability and self-efficacy to deliver equitable, respectful, high-quality care, which reinforced trust relationships with patients. Our findings, remarkable given the 9-fold increase in curative caseload and escalating security crisis, contribute to evidence that links health systems support, trust, respect, motivation and performance of health workers (Okello and Gilson, 2015; Munabi‐Babigumira *et al*., 2017), including CHWs (Glenton *et al*., 2013; Kok *et al*., 2015a; 2015b; Scott *et al*., 2018). Participants’ perceptions and expectations of the quality of healthcare, rooted in their experiential learning and social networks, drive child (Colvin *et al*., 2013; Scott *et al*., 2014) and maternal (Freedman and Kruk, 2014) utilization in other disadvantaged contexts. We contribute novel findings about speed to care: via multiple pathways to impact, ProCCM engendered a context of facilitated access, quality care and prompt utilization, as participants sought curative child healthcare faster and preventive maternal healthcare earlier. Across trial arms, 24-hour treatment among children more than doubled (Whidden *et al*., 2023) and first trimester ANC increased by 15% (Kayentao *et al*., 2023).

Proactive CHW home visits triggered mechanisms that were already activated in both trial arms, which explains the modest improvements in utilization and no effect on under-5 mortality attributable to home visits. First, doorstep care further reduced distance and opportunity costs to CHW services, enabling marginalized participants who faced poverty, time constraints, gendered social norms and/or limited mobility to make/realize healthcare choices. This process evaluation indicated that, in the intervention arm compared with control, more sick children were assessed by CHWs, assessed earlier, had less severe symptoms and were followed up more. In our trial outcome evaluation, sick children in intervention clusters were more likely to receive healthcare overall compared with control (Whidden *et al*., 2023), and subgroup analyses suggested that home visits may have improved child access to care most in remote communities and the poorest households (Whidden *et al*., 2023). CHW home visits have been found in other contexts to have pro-equity effects (McCollum *et al*., 2016; Schleiff *et al*., 2017; Blanchard *et al*., 2019). Second, home visits helped CHWs build relationships, trust and social capital (Kane *et al*., 2020; Schaaf *et al*., 2020; Ndambo *et al*., 2022), and patients learn about quality of healthcare and what they should expect. As these processes take time (Leonard, 2014), home visits may have made a difference in curative care utilization at the beginning of the programme, while feedback loops (Marchal *et al*., 2013) and social networks via participants’ own and shared experiences sustained and ultimately overtook its effects. In the trial, children were more likely, due to home visits, to receive prompt treatment at 12 months but not thereafter (Whidden *et al*., 2023). Home visits may also have more effect via these relational and experiential mechanisms on early preventive or complete follow-up care (Gilmore and Mcauliffe, 2013; Wroe *et al*., 2021; Yonemoto *et al*., 2021) than time to treatment. The trial found 11% and 25% increases in first trimester ANC and four or more ANC, respectively, in the intervention arm compared with control (Kayentao *et al*., 2023), and CHW home visits during pregnancy have improved ANC attendance in other contexts (Edmond *et al*., 2018; Katzen *et al*., 2020).

Although we quantified home visits conducted by CHWs (Supplementary Figures S3 and S4), we were unable to measure fidelity to the workflow protocol at the household level: at least two home visits per household per month in the intervention arm and no home visits per household per month in the control arm, continuously throughout the trial. IDIs suggested good adherence to the CHW workflow, but survey responses from the ProCCM trial indicated that only 47% and 78% of child-year observations in intervention and control arms, respectively, met the per protocol definition in the preceding month. The trial’s per protocol analyses suggested that, while poor adherence may partially account for the subdued effects on child healthcare utilization between arms (Whidden *et al*., 2023), they do not explain the null effects of home visits on under-5 mortality (Liu *et al*., 2024). Our forthcoming dose-response analysis aims to generate a reliable denominator between CHW mobile application data and trial survey data and assess the relationship between home visit ‘dose’ and mortality outcome. Nevertheless, this process evaluation shows how the ProCCM trial’s null main effects are due, at least in part, to the co-interventions and overlapping mechanisms across both trial arms. Poor adherence in intervention arms and ‘exceptional’ services in control arms, which overlap with and dilute the primary interventions being tested, have been found to explain null results of other trials (Padian *et al*., 2010).

We note that IDIs with participants and providers were overall positive about ProCCM, and we need to conduct further investigation to better understand how or why many children still did not access care or died during the trial. Some respondents could have been inclined to give biased responses out of loyalty to their CHW (such as how frequently their proactive CHW visited their home) or to ensure the programme continued. Furthermore, power imbalances could have come into play between interviewers and respondents, intimidating some respondents and hindering collaborative theory refinement. Interviewers used traditional qualitative interview techniques of building rapport, body language, tone and active listening to put respondents at ease, and we only incorporated realist interviewing techniques after asking open-ended questions (Gilmore, 2019). We also observed that some respondents contradicted initial programme theories that we put to them, including reactions of female respondents to theories that had to do with gender. Some of our tentative theories were not understood well by respondents, and we considered this to be evidence that the theory did not resonate, which helped us refine our overall CIAMOs. We noted that the use of translators during some interviews could have led to misunderstandings or a loss of information or nuance, and it would have strengthened our study had we involved translators directly in the interpretation of data and consolidation of theories (Gilmore, 2019). Finally, although we consider the two rounds of IDIs a strength of this evaluation, we lacked baseline interviews, which is an important limitation given how central context was in this evaluation and is in realist evaluations more broadly. However, we were able to capture important elements of the baseline context by asking questions about changes.

CHW interventions need to be evaluated with frameworks that address complexity inherent in community health systems. Trialling individual components in isolation, like CHW home visits, may not reflect real life programme implementation or accommodate multiple components working together in non-linear ways (Hargreaves *et al*., 2019). In this process evaluation, we were able to explain ProCCM trial results between and across arms, and generate ProCCM programme theories that link outcomes to contexts and mechanisms, by combining ToC and realist approaches and embedding them within a process evaluation framework. We propose a cascade of CIAMO nodes that interact within and between each other to hold the interplay between multiple ProCCM components together, centre the expertise of both providers and participants as actors who interpret and construct health systems and reflect the dynamic, non-linear processes that are healthcare-seeking decisions. Although the changes in outcomes across trial arms compared with baseline are observational results, this process evaluation contributes to the plausibility that ProCCM led to these improvements, which specific components drove effects and how. We treated context as dynamic, which interacted with the implementation process, activated mechanisms (or not) and affected outcomes in our trial. Thus, our empirical theories can be used to elaborate mid-range theories that can be tested in other contexts to consider the transferability of ProCCM and CHW home visits (Nilsen, 2015).

## Conclusion

ProCCM’s user fee removal, professional CHWs and upgraded PHCs in both trial arms accelerated access to healthcare and cut under-5 mortality by more than a half via multiple pathways to impact that interacted in complex ways with both structural barriers and people’s agency, and reshaped the broader health system and social context. In the intervention arm, proactive CHW home visits prompted increased, rapid child and maternal healthcare utilization via similar mechanisms, thus diminishing expected effects of this singular component. Our findings contribute to research and policy discussions on how to design, implement and evaluate community health systems that support CHWs, serve the most marginalized and optimize impact and learning.

## Supplementary Material

czae066_Supp

## Data Availability

The data underlying this article will be shared on reasonable request to the corresponding author with permission of Muso, and of the Government of Mali in the case of DHIS2 data.

## Abbreviations

ANC = antenatal care

ASACO = community health association

CI = confidence interval

CIAMO = context-intervention-actor-mechanism-outcome

CHW = community health worker

DHIS2 = District Health Information Software II

IDI = in-depth interview

PHC = primary health centre

ProCCM = proactive community case management

ToC = theory of change

## Reflexivity statement

Over half of the 19 authors are Malian researchers, including the second and penultimate-senior authors. Five authors are female, including the first, third and senior authors, but none of the female authors are Malian and this is a critical gap. Six authors are early career researchers (three female and three male) and these authors led data analysis. The authors reflect a range of expertise and contributions to this research.

We have prepared a longer author reflexivity statement that we would like to include as a supplementary file if our paper is accepted, but we have left it out of the submission to protect Health Policy and Planning’s double anonymized review process.
