# Supplementary material for: Community case management to accelerate access to healthcare in Mali: a realist process evaluation nested within a cluster randomized trial
Source: Health Policy Plan. 2024 Jul 26;39(8):864–77. doi: 10.1093/heapol/czae066 (PMC11384120; doi:10.1093/heapol/czae066)
Supplement: czae066_Supp [file czae066_supp.zip › R&R Process Evaluation - Supplementary Figures v1Aug2023 Clean.docx]

# Supplementary figures

Figure S1: The ProCCM trial design


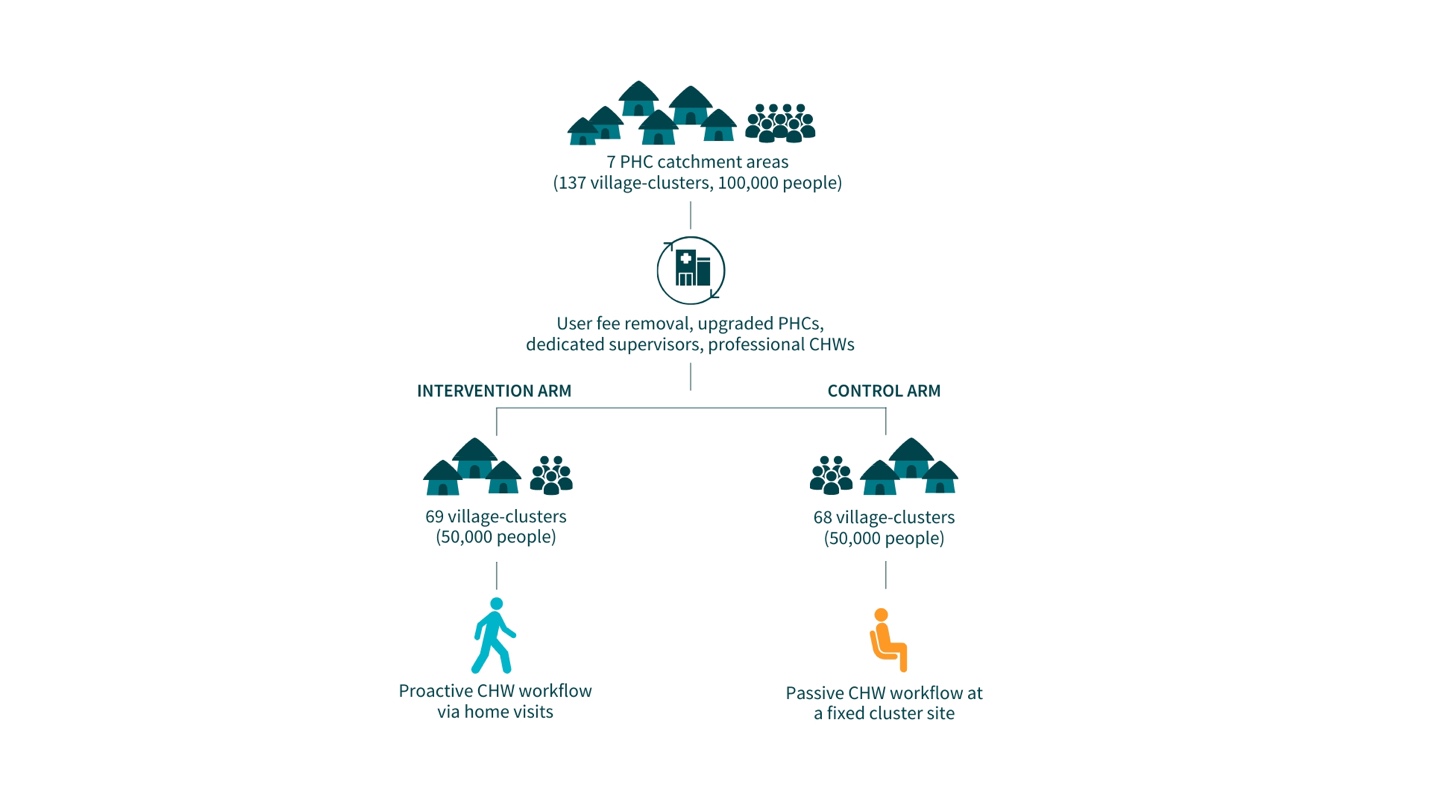


Figure S2: Number of first antenatal care visits at PHCs during the 14 months prior to ProCCM launch and the trial period


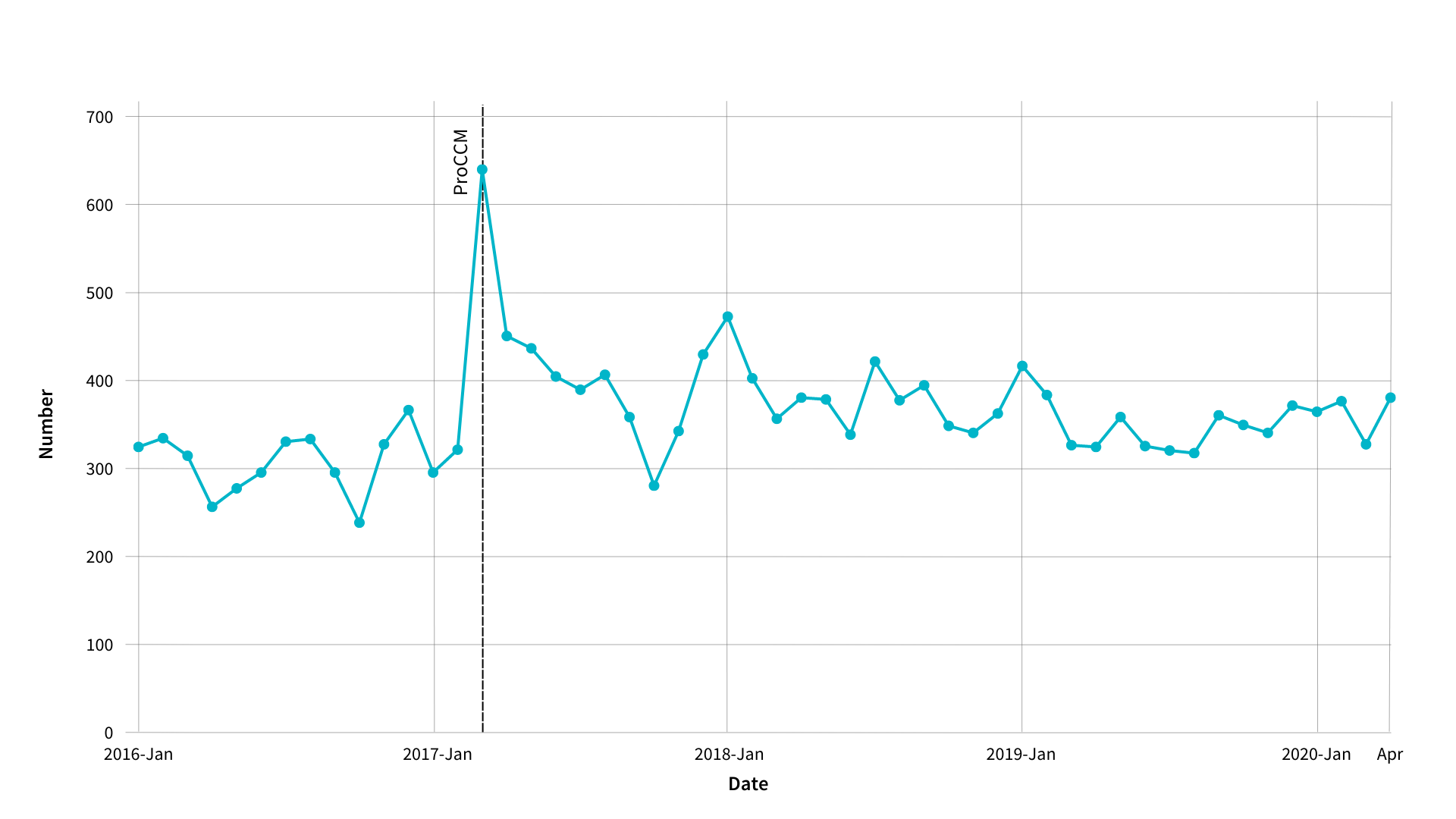


Data source: DHIS2. Notes: the monthly mean in the trial period is 378 first ANC visits, and in the pre-programme period is 309. At two-sample t test with equal variances yields a statistically significant (p<0.0001) difference between the two means. Figure S3: Proactive CHW home visits per month by trial arm

Notes: Data comes from the CHW application and is available starting only in September 2017 (7 months into the trial) when the home visit form was integrated into the application.

Figure S4: Box plot of total proactive CHW home visits per month by trial arm

Notes: 30 months of data available from the CHW application

Figure S5: Share of CHW new curative consultations/sick patient assessments among under fives conducted the same or next day of symptom onset, per month by trial arm

Source: CHW application

Figure S6: Share of sickness among under fives assessed by CHWs, per month by trial arm

Source: CHW application

Figure S7: Number of treatment and referral follow up visits by CHWs, per month by trial arm

Source: CHW application
